# Supplementary material for: Transcriptome Profile of Membrane and Extracellular Matrix Components in Ligament-Fibroblastic Progenitors and Cementoblasts Differentiated from Human Periodontal Ligament Cells
Source: Genes (Basel). 2022 Apr 8;13(4):659. doi: 10.3390/genes13040659 (PMC9031187; doi:10.3390/genes13040659)
Supplement: Supplementary file 1 [file genes-13-00659-s001.zip › Supplementay data-RNASeq.pdf]

< Supplementary Results >

Transcriptome profiles of membrane/extracellular matrix components in two different progenitors differentiated with TGF-β1 and BMP7 from human periodontal ligament cells

Seyoung Mun,[1] Seong Min Kim,[1] Min-Jeong Choi,[1] and Young-Joo Jang[1, 2,*]

[1]Department of Nanobiomedical Science & BK21 FOUR NBM Global Research Center for Regenerative Medicine, Dankook University, Cheonan, 31116 Korea, [2]Department of Oral Biochemistry, School of Dentistry, Dankook University, Cheonan, 31116 Korea

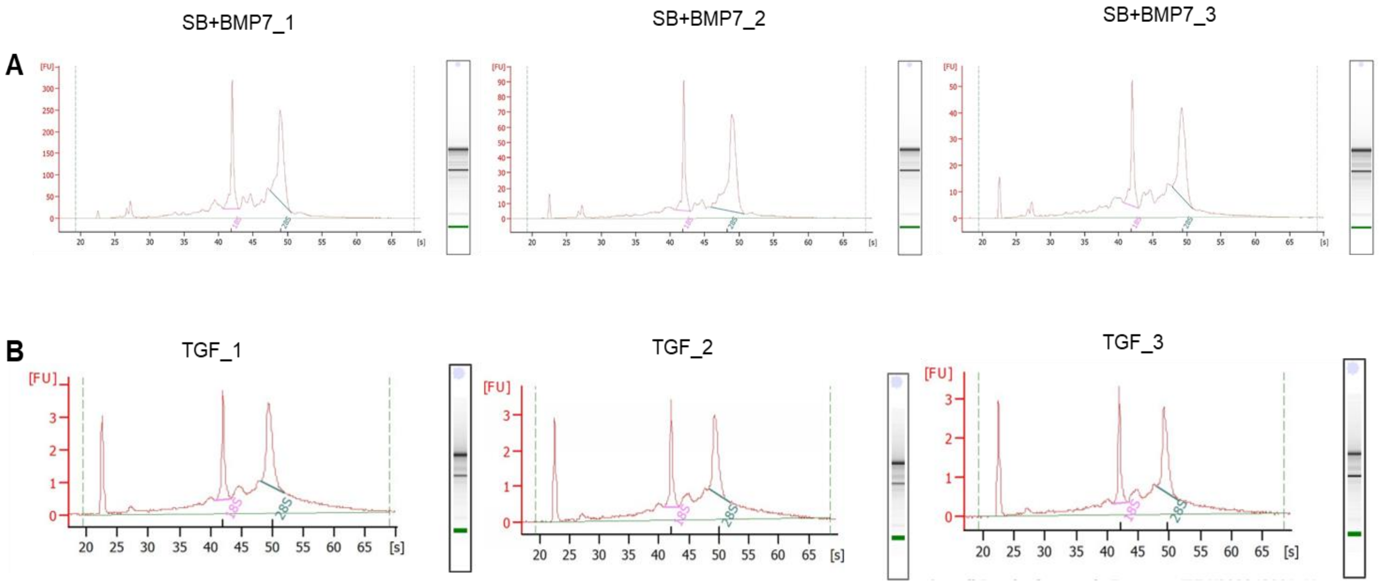

Supplementary Figure 1. Experimental data for qualifying the RNA samples from the groups treated with SB+BMP7 (A) and TGF-β1 (B). Size distribution analysis of each sample was obtained using an Agilent 2100 Bioanalyzer instrument. All proper size of RNA sample were prepared.

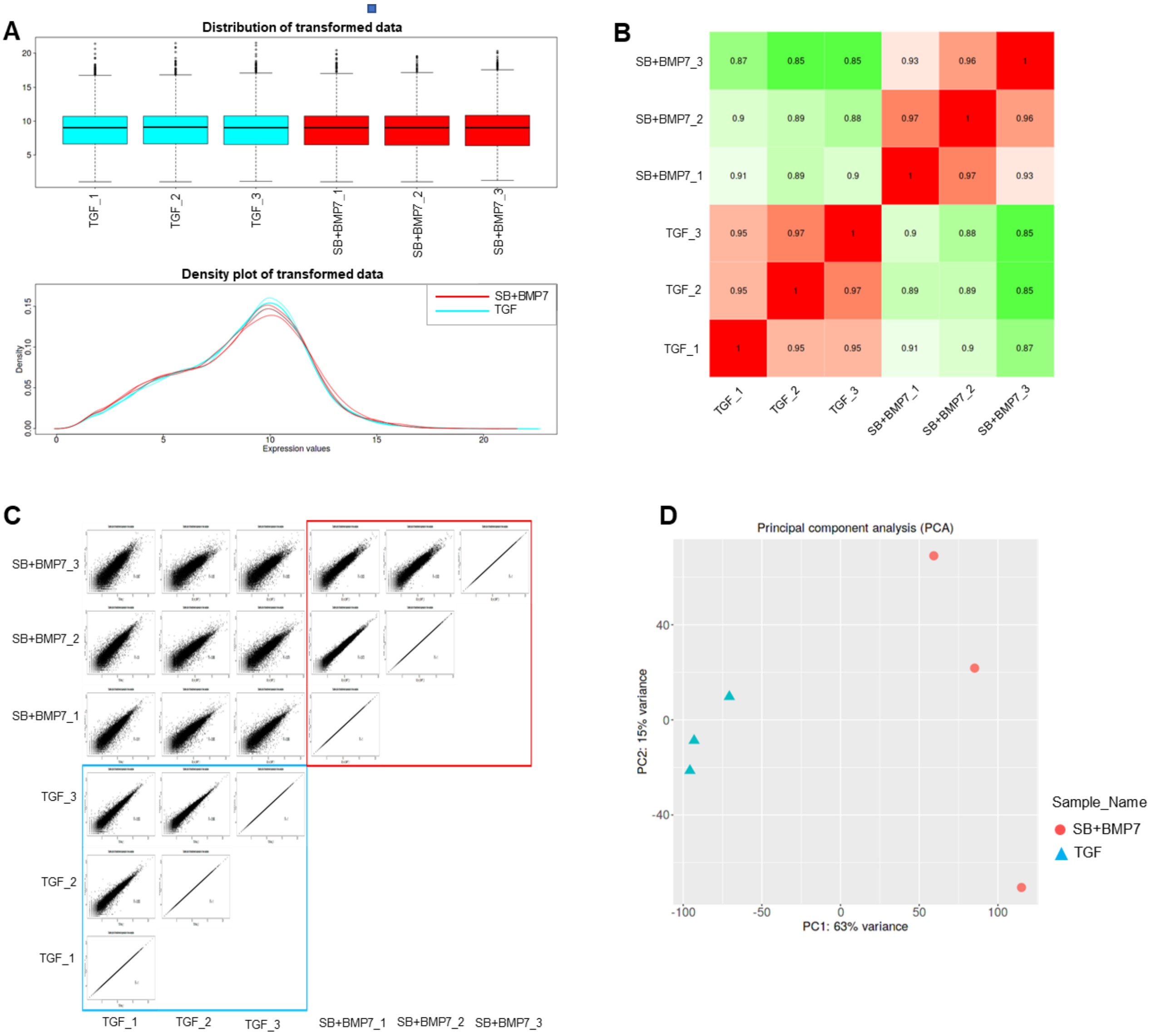

Supplementary Figure 2. Bioinformatical observation for expression data and sample correlation. (A) The boxplot showed the gene expression of each sample subsequent to data normalization. The ordinate represented gene expression value, and the abscissa represented the groups of SB+BMP7- and TGF-β1-treated cells according to the emerald and red color. Distribution of FPKM values for total expressed genes in samples of each group is also shown. (B) The Euclidean distances based on all raw expression data, were calculated between each sample and the colors indicating the distances. (C) Scatter plot graphs of comparative expression profiling between six samples. The scatter plot indicated the difference in mRNAs expression in the groups of SB+BMP7 and TGF-β1 treatment. (D) The MDS plot showed how similar and close the transcriptome changes of each sample are based on the global gene expression level.

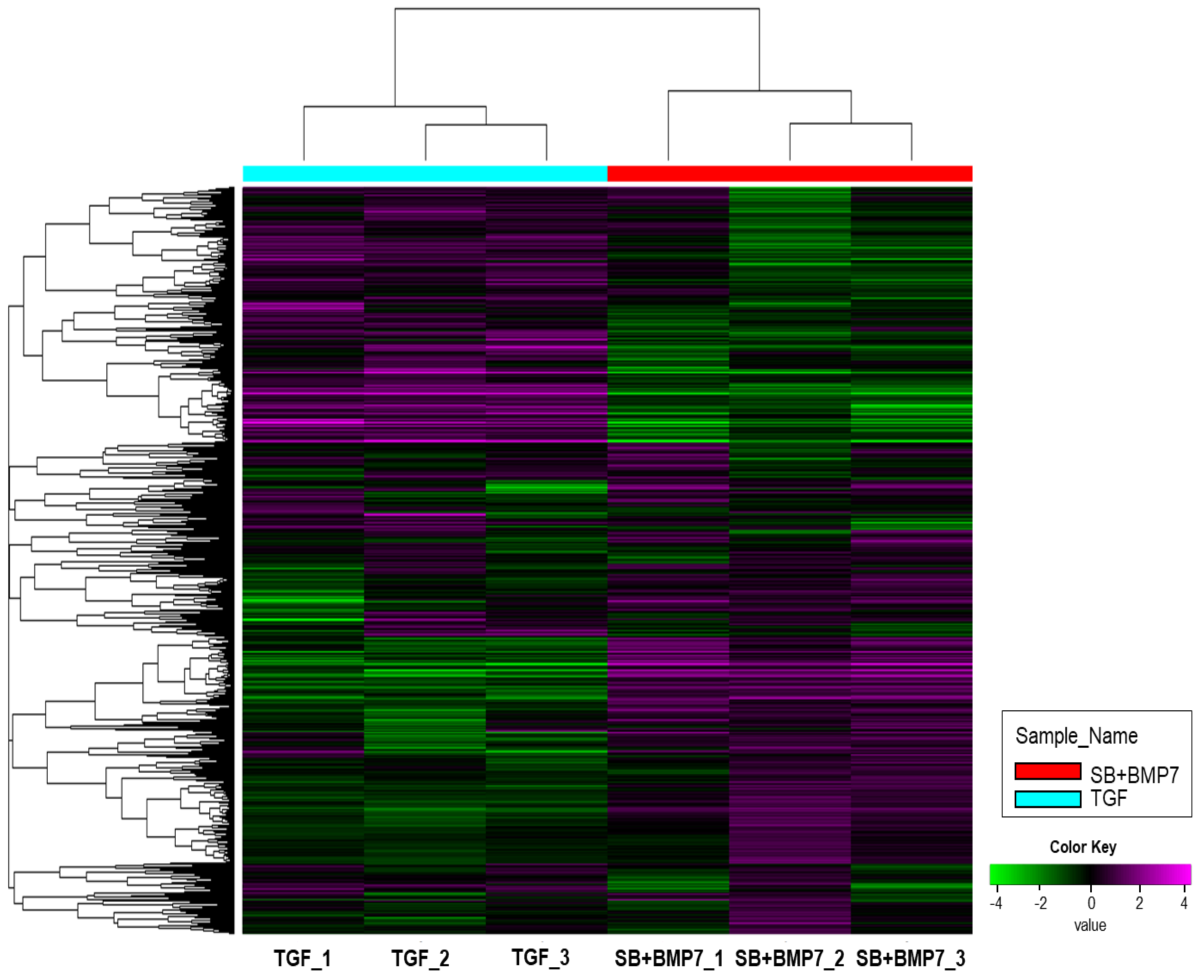

Supplementary Figure 3. Global expression for total expressed genes. Hierarchical clustering analysis of total expressed genes was shown. A histogram in the bottom color key indicated number of expression values within color bar.

Supplementary Figure 4. Original blots for the results in Figure 7.

(A) Blots for Figure 7A (the representative genes upregulated by TGF-β1 treatment)

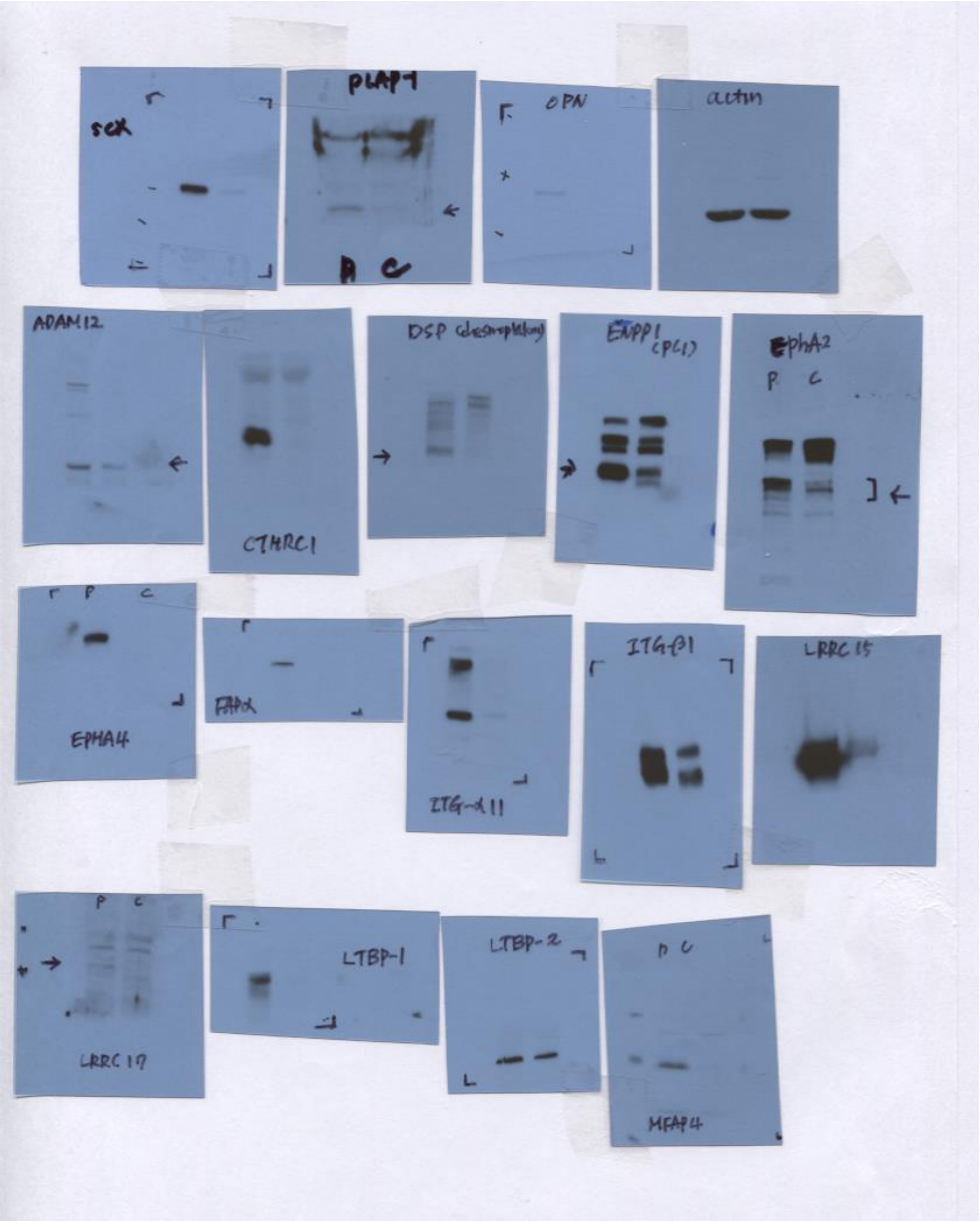

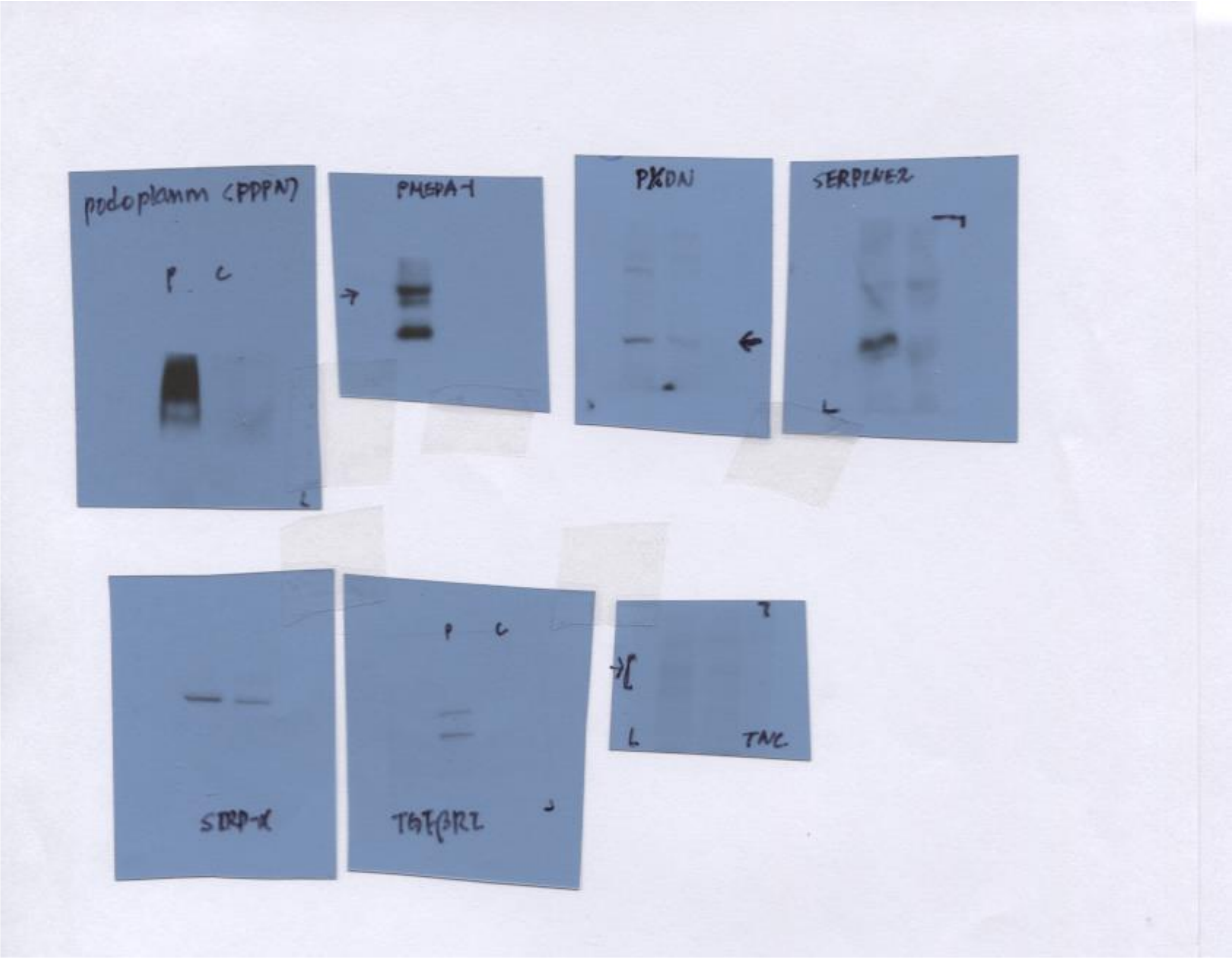
podoplanin (PDPN)
P C
PMEPA-1
SERPINE2
P C
TGFβR2
TNC

Supplementary Figure 4. Original blots for the results in Figure 7.

(B) Blots for Figure 7B (the representative genes upregulated by SB/BMP7 treatment)

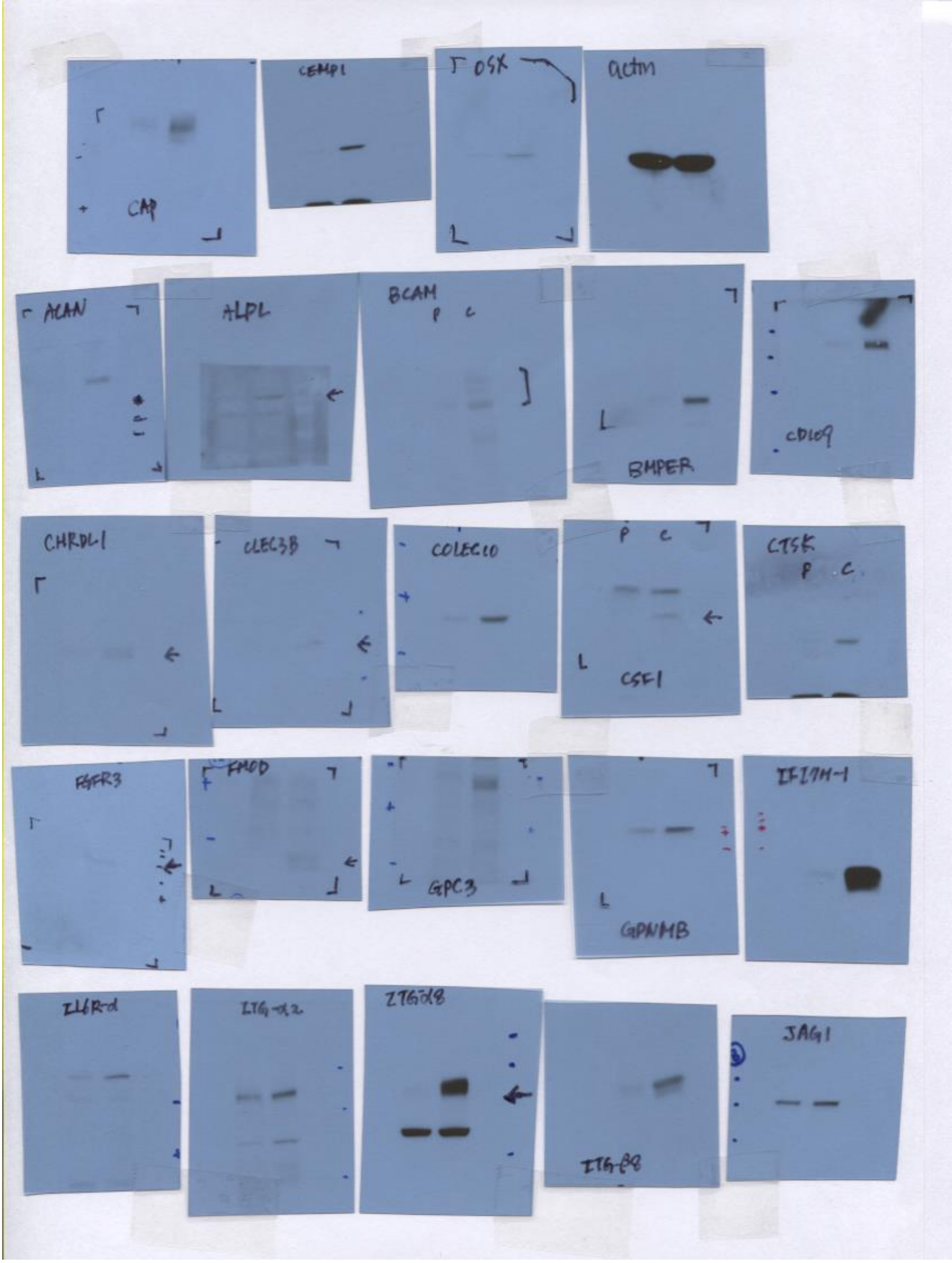

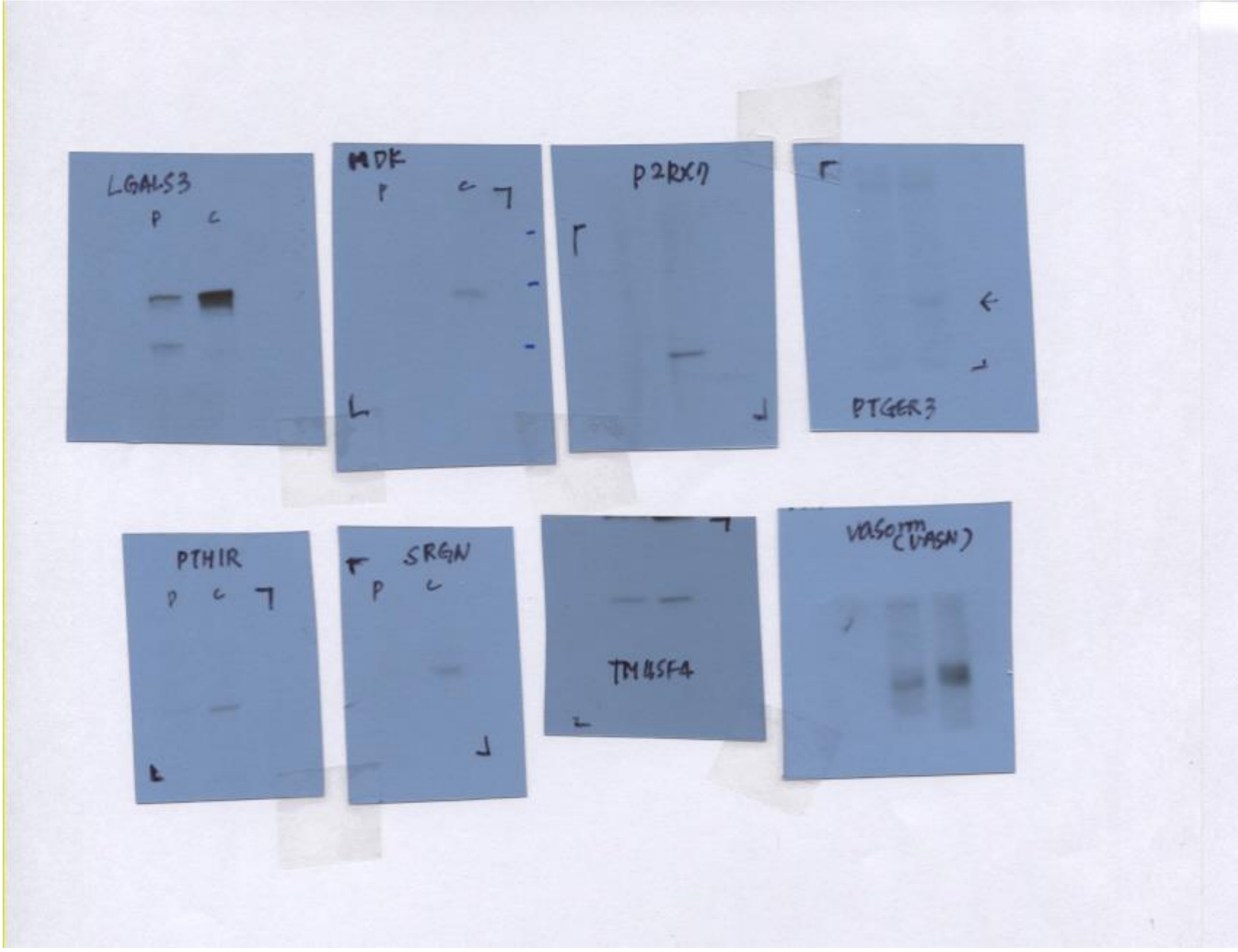
LGALS3
P C
MDK
P C
P2RX7
PTGER3
PTH1R
P C
SRGN
P C
TM4SF4
Vasorin (VASN)
